# Pharmacological considerations in the design of anti-malarial drug combination therapies – is matching half-lives enough?

**DOI:** 10.1186/1475-2875-13-62

**Published:** 2014-02-20

**Authors:** Ian M Hastings, Eva Maria Hodel

**Affiliations:** 1 Liverpool School of Tropical Medicine, Pembroke Place, Liverpool L3 5QA, UK

**Keywords:** Antimalarials, Combination therapy, Pharmacokinetics, Pharmacodynamics, Drug resistance

## Abstract

Anti-malarial drugs are now mainly deployed as combination therapy (CT), primarily as a mechanism to prevent or slow the spread of resistance. This strategy is justified by mathematical arguments that generally assume that drug ‘resistance’ is a binary all-or-nothing genetic trait. Herein, a pharmacological, rather than a purely genetic, approach is used to investigate resistance and it is argued that this provides additional insight into the design principles of anti-malarial CTs. It is usually suggested that half-lives of constituent drugs in a CT be matched: it appears more important that their post-treatment anti-malarial activity profiles be matched and strategies identified that may achieve this. In particular, the considerable variation in pharmacological parameters noted in both human and parasites populations may compromise this matching and it is, therefore, essential to accurately quantify the population pharmacokinetics of the drugs in the CTs. Increasing drug dosages will likely follow a law of diminishing returns in efficacy, i.e. a certain increase in dose will not necessarily lead to the same percent increase in efficacy. This may allow individual drug dosages to be lowered without proportional decrease in efficacy, reducing any potential toxicity, and allowing the other drug(s) in the CT to compensate for this reduced dosage; this is a dangerous strategy which is discussed further. Finally, pharmacokinetic and pharmacodynamic drug interactions and the role of resistance mechanisms are discussed. This approach generated an idealized target product profile (TPP) for anti-malarial CTs. There is a restricted pipeline of anti-malarial drugs but awareness of pharmacological design principles during the development stages could optimize CT design pre-deployment. This may help prevent changes in drug dosages and/or regimen that have previously occurred post-deployment in most current anti-malarial drugs.

## Background

The benefits of using a combination of drugs to treat infectious diseases has been recognized since the days of Laveran [1] and Ehrlich [2] who used combination therapy (CT) with dyestuffs to treat trypanosomiasis. Drug CTs are now standard policy for treating human immunodeficiency virus (HIV) infections, tuberculosis (TB) and malaria. In the case of TB and HIV, the use of CT was driven by clinical necessity as patients routinely failed treatment with monotherapies. The use of CTs to treat falciparum malaria, currently in the form of artemisinin-based combination therapy (ACT), rests on a different justification. Most of the partner drugs in ACT are clinically effective as monotherapies, especially when first deployed, so the use of anti-malarial CTs is primarily justified as a public health strategy to delay or even prevent the onset of resistance (e.g. [3], recently reviewed in [4]). There is considerable concern about the possible onset of artemisinin resistance, particularly in South-East Asia [5-9] and its long-term threat to the future of ACT (e.g. [10-14]). Several new anti-malarial drugs are in the developmental pipeline [15,16] so new forms of CT will have to be considered. Herein, the design principles underlying anti-malarial CTs are discussed. Clearly identifying desirable properties of constituent drugs in an anti-malarial CT may well help guide the developmental pathway and stop/go decision-making process of anti-malarials currently under development [17].

Previous models demonstrating the benefits of CT in delaying the onset of resistance largely assumed that parasites are either completely sensitive to the drug, or else completely resistant (discussed further in [4]). The first type of model, the ‘basic rationale’ [4] considers how new mutations enter the malaria population. Assuming the mutation rate to resistance to each drug is 10^-9^, then the chance of any individual parasites being spontaneously resistant to both drugs in a CT is vanishingly small (i.e. 10^-18^). Thus, new mutations should enter the parasite population extremely rarely (note, however, the implicit assumption that resistant mutations only enter the population through drug treatment of the asexual biomass of typically about 10^11^ to 10^12^ parasites per patient [18]; discussed in more detail in [4]). The second type of model investigates the dynamics of resistance once it has entered the malaria population. For example, models of a two-drug CT typically assume that resistance to each drug is encoded by a different, single locus with alleles classified as resistant (R) and sensitive (S). This gives rise to four 2-locus haplotypes that can be designated S_A_S_B_, R_A_S_B_, S_A_R_B_ and R_A_R_B_, where subscript denotes resistance status to drug ‘A’ or ‘B’ respectively. Consequently, infections of types S_A_S_B_, S_A_R_B_ and R_A_S_B_ are always killed by CT treatment while only R_A_R_B_ survives. This strict dichotomy of ‘resistant’ versus ’sensitive’ forms is useful for elucidating the general principles underlying CT design but is not a particularly realistic representation of treatment outcome which is known to depend not just on parasites genotype, but on other factors such as immunity and drug dosage [19]. In addition, these types of models can only demonstrate the broad benefits of CT but they cannot provide information about the optimal designs of these CTs. The subtleties of how resistance evolves may be complex (for example, artemisinin resistance may be restricted to specific parts of the parasite life cycle [20]) and are discussed elsewhere [4,21]. This manuscript will ignore how resistance actually arises, will simply assume that it is inevitable, and will focus on considering how pharmacological modelling can help design CTs that are robust to increasing levels of parasite resistance entering the parasite population.

A more nuanced approach to resistance/sensitivity can be achieved using pharmacokinetic/pharmacodynamic mechanism-based modelling of drug treatment (recently reviewed in [22]). In this context of the pharmacology of malaria treatment, pharmacokinetics (PK) describes how drugs are processed by the human body, e.g. the drug half-life, while pharmacodynamics (PD) describes how the drug affects the parasite e.g. the drug concentration producing half the desired effect (IC50). There are potentially several different mechanisms and models of PD depending on how the drug acts: some drug actions may best be described by the maximum concentration reached, the times above a certain concentration, or the extent to which a drug accumulates at the target site. A recent review and access to the literature is provided in [23] but here, for simplicity, the discussion assumes anti-malarial drugs can be best described by standard modelling [22] as previously applied to malaria drug action (see below). The technique can be summarized as follows. The drug concentration profile after treatment is tracked using PK modelling while the sensitivity of the parasites to the drugs is defined by its PD parameters. The drug concentration at any time post-treatment can, therefore, be translated into a parasite kill rate enabling change in parasites numbers post-treatment to be tracked in order to find whether the parasites are eventually eliminated, or whether they survive treatment. Critically, this strategy allows researchers to define ‘resistant’ parasites in mechanistic, PD, terms such as increased IC50 rather than simply assuming they are completely insensitive to the drug. The fate of the ‘resistant’ parasites can then be investigated in the context of all the other factors known to affect patient outcome; typical examples are the pharmacological environment parasites encounter during treatment (derived from varying patient PK parameters, e.g. drug elimination rate), the patient’s adherence to treatment and so on. This approach has already been applied to malaria [24-27] and recent work [28-30] has focussed on developing the technical methodology to allow for multiple drug doses, combination therapies and drug conversion processes. A parallel data-driven agenda has investigated the nature and extent of variation in the PK/PD parameters and extension to real-life deployment such as age- or weight-based dosing bands and the impact of poor adherence to the recommended regimens [30]. Unlike models based on sensitive/resistance dichotomy, these PK/PD models do throw light on what properties define a good CT and herein it is argued that PK/PD considerations can usefully contribute to the rational design of CTs.

### Key pharmacological considerations for potential combination therapies

There are six distinct considerations that must be addressed in the design of CT which will each be addressed in turn.

#### 1. The half-lives and activity profiles of constituent drugs

**Figure 1:**
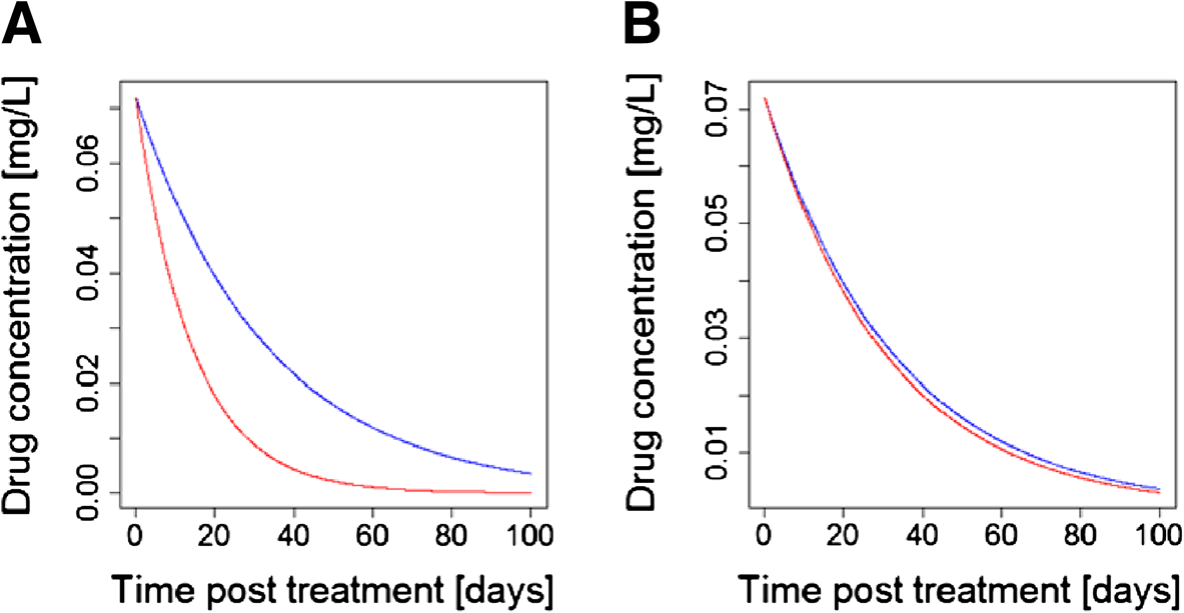
**The consensus view that drugs in a CT should have matching half-lives [**[32]**,**[33]**]. (A)** The constituent drugs have very different half-lives (as in the current generation of ACT) leaving the ‘blue’ drug to persist as a vulnerable monotherapy for an extended period of time post-treatment after the ‘red’ drug concentration has decayed to sub-therapeutic concentrations. **(B)** The constituent drugs have roughly similar half-lives meaning they should, in principle (but see main text), provide mutual protection post treatment. [Figure 1 was constructed using simple PK/PD models and their corresponding equations [26,29]. Parameter values for the two drugs were as follows: Dose is 11 mg/kg; volume of distribution is150 L/kg. Elimination rates per day were 0.03 for ‘blue’ and 0.07 for ‘red’ (equivalent to half-lives of 23.1 and 9.9 days, respectively) in **(A)** changing to 0.032 for ‘red’ in **(B)** (equivalent to half-life of 21.7 days)].

It has long been realized [31] that a mismatch in half-lives will leave the drug with the longer half-life to persist as a vulnerable monotherapy (Figure 1) because new infections emerging from the liver (or inoculated by the mosquito in the case of drugs that are active against liver stages such as atovaquone) need only be resistant to a single drug to survive. This effect constitutes one of the three main drivers of anti-malarial drug resistance [4]. Therefore, a key recommendation in CT design is that the drug half-lives should be matched (e.g. [32]) but this argument warrants further examination.

**Figure 2:**
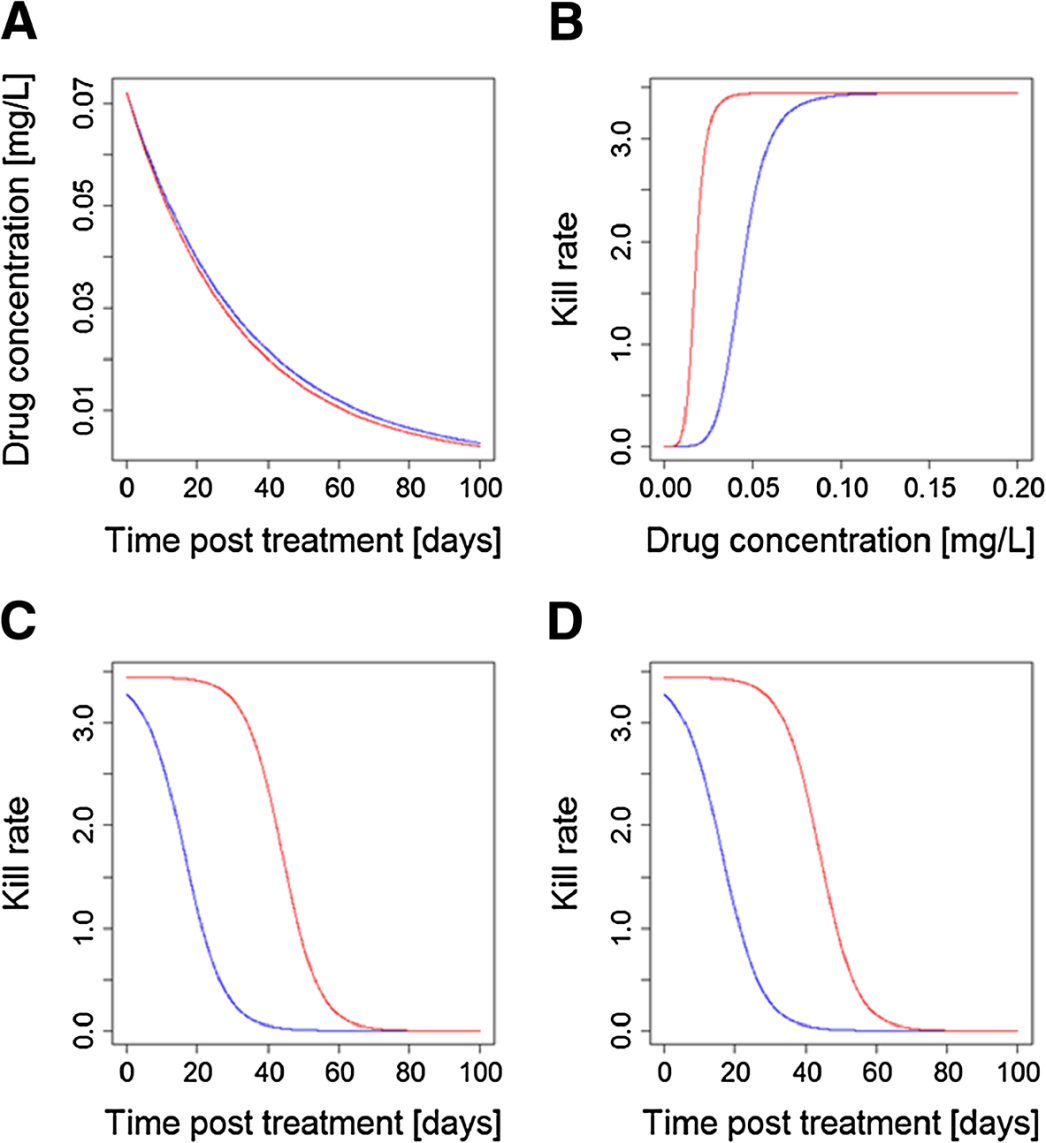
**Is it more important to match post-treatment activity profiles rather than crude drug half-lives? (A)** Two drugs in a CT have broadly similar half-lives. **(B)** The two drugs in the CT have very different PD profiles. **(C)** Multiplying concentration profiles post-treatment (shown in **(A)**) by the dose-effect relationships (shown in **(B)**) gives a drug activity profile post-treatment; as can be seen these profiles are very different leading one drug to persist as a vulnerable monotherapy. **(D)** A practical example of this effect: the drugs appear to be perfectly ‘matched’ with similar half-lives (as in **(A)**) and identical kill rates (both assumed to have the ‘blue’ profile shown in **(B)**), but toxicity concerns means the ‘blue’ drug must be given at 2.5-fold lower dosages, leading to a severe mismatch in drug activity profiles. Note the similarities between the results shown in **(C)** and **(D)**. [Figure 2 was constructed using simple PK/PD models and their corresponding equations [26,29]. Parameter values for the two drugs are as follows: Dose is 11 mg/kg for both in **(A)**, **(B)** and **(C)** and 11 mg/kg for ‘blue’ and 27.5 mg/kg for ‘red’ in **(D)**; volume of distribution is 150 L/kg; elimination rate per day is 0.03 for ‘blue’ and 0.032 for ‘red’ (equivalent to half-lives of 23.1 and 21.7 days, respectively); maximal drug-killing rate per day (Vmax) is 3.45; IC50 is 0.044 mg/L for ‘blue’ and 0.0176 mg/L for ‘red’ in **(A)**,**(B)** and **(C)** and 0.044 mg/L for both in **(D)**; slope of dose-response curve (n) is 6].

This argument is couched in terms of half-life but matching is more subtle because mutual protection between the drugs depends on there being *active* concentrations of both drugs simultaneously. It, therefore, appears more important that drug anti-malarial activity profiles post-treatment are the critical factors to be matched, rather than simply their half-lives. The activity profile post-treatment depends on three factors, the drug half-lives, the dosage given (and absorbed) and the drugs’ endogenous anti-malarial activity. Figure 2 shows how matched half-lives may be undermined by differences in anti-malarial drug activity and/or if concerns over toxicity mean the drugs must be given at different dosages; either situation will result in a period of time where only one drug is responsible for killing parasites and so is in effect a monotherapy.

**Figure 3:**
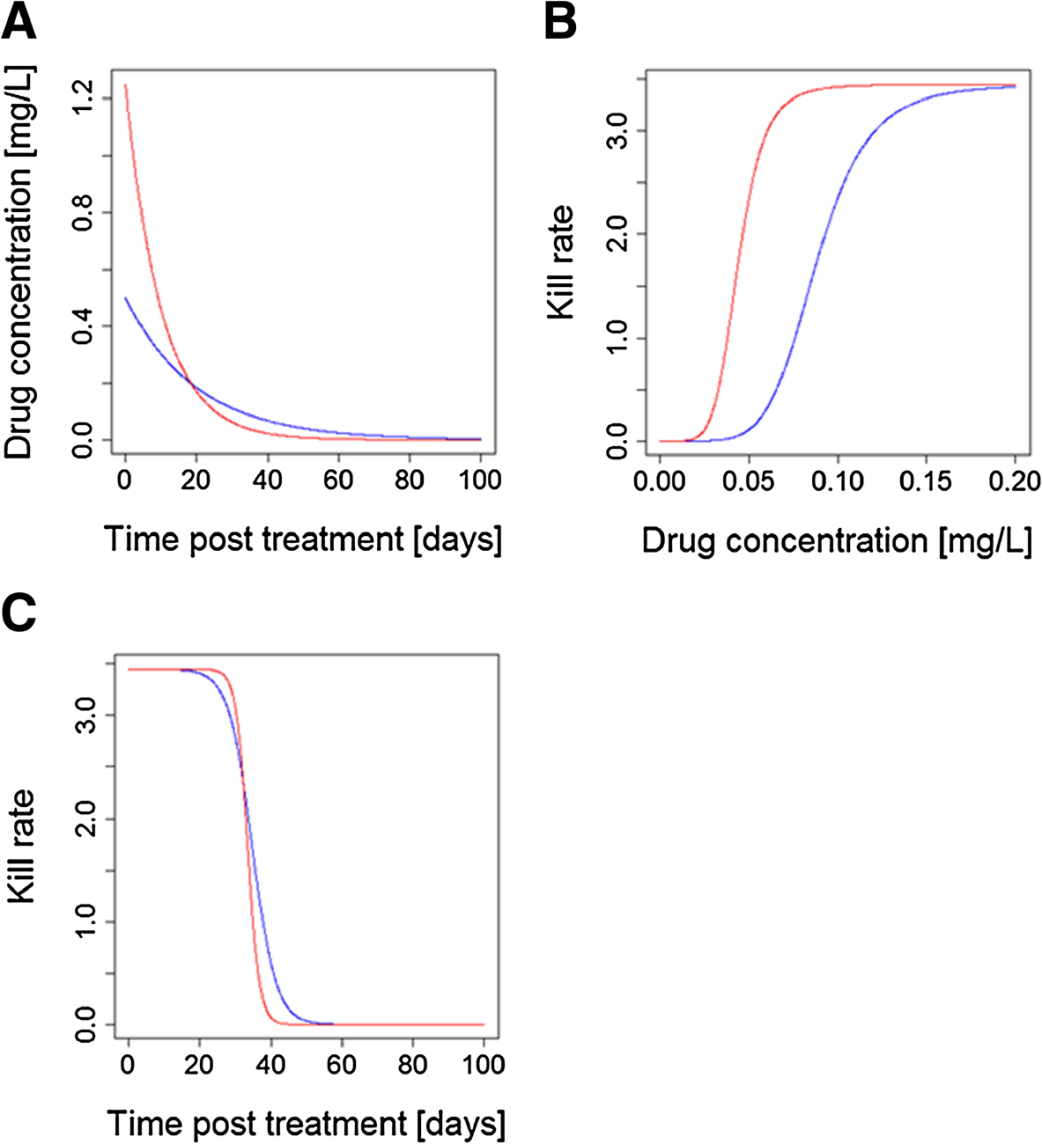
**How altering relative dosages can compensate for differences in half-live and/or endogenous anti-malarial activity. (A)** The half-lives of two drugs in this CT differ by a factor of 2, leading to one drug being left as a vulnerable monotherapy; most arguments on design of CT end here by concluding the drugs are not well matched. **(B)** The drug kill rates against parasites as a function of drug concentration; they differ in their IC50 values. **(C)** Compensating for differing half-lives and IC50s by increasing the dosage of drug illustrated in ‘red’ 2.5 fold: killing is now matched and drugs provide mutual protection. [Figure 3 was constructed using simple PK/PD models and their corresponding equations [26,29]. Parameter values for the two drugs are as follows: Dose is 75 mg/kg for ‘blue’ and 187.5 mg/kg for ‘red’; volume of distribution is 150 L/kg; elimination rate per day is 0.05 for ‘blue’ and 0.1 for ‘red’ (equivalent to half-lives of 13.8 and 6.9 days, respectively); maximal drug-killing rate per day (Vmax) is 3.45; IC50 is 0.088 mg/L for ‘blue’ and 0.044 mg/L for ‘red’; slope of dose-response curve (n) is 6].

One way in which matching of drug killing can be quantified is by their time above the minimal inhibitory concentration (MIC) a concept originally used in bacteriology but now being increasingly used in malaria (e.g. [34] for a recent example). Time above MIC for any anti-malarial drug is a function of the maximum concentration (Cmax) and drug clearance, which are again dependent on the dose and the formulation, and the MIC, which is described by the PD profile. More encouragingly, the interaction between factors such as half-life, dosage and parasite drug sensitivity means that a well-designed CT could, in principle, allow for mismatch in any of these variables by altering the relative dosages (Figure 3) to achieve matched activity profiles post-treatment. This does raise the interesting, and largely unconsidered, operational question of whether both drugs should be deployed in a CT at their maximum dosages, or whether one drug may be included at a reduced dosage to match killing activities. The former is probably more robust. Maximal concentrations of both drugs optimize clinical effectiveness and help protect against resistance being driven through drug failures (the ‘matching’ argument only applies to selection for resistance post-treatment [35]) and natural variation in PK/PD may, in practice, largely undermine matching done on average PK/PD values (see below). It is difficult to bring toxicity arguments into this discussion at present because this is currently an under-researched area (see below). The onset of toxicity associated with anti-malarial drugs is generally not indicated by clinically clear on/off signals and, moreover, an increased risk of adverse events does not necessarily follow a direct correlation with plasma exposure (either area under the curve, AUC, or Cmax).

In summary, although it is essential to reiterate the usual assertion that matching half-lives is important we suggest it should be seen in a more subtle way and that activity profiles after treatment are of more importance than crude half-lives.

#### 2. Natural variation in population PK/PD

**Figure 4:**
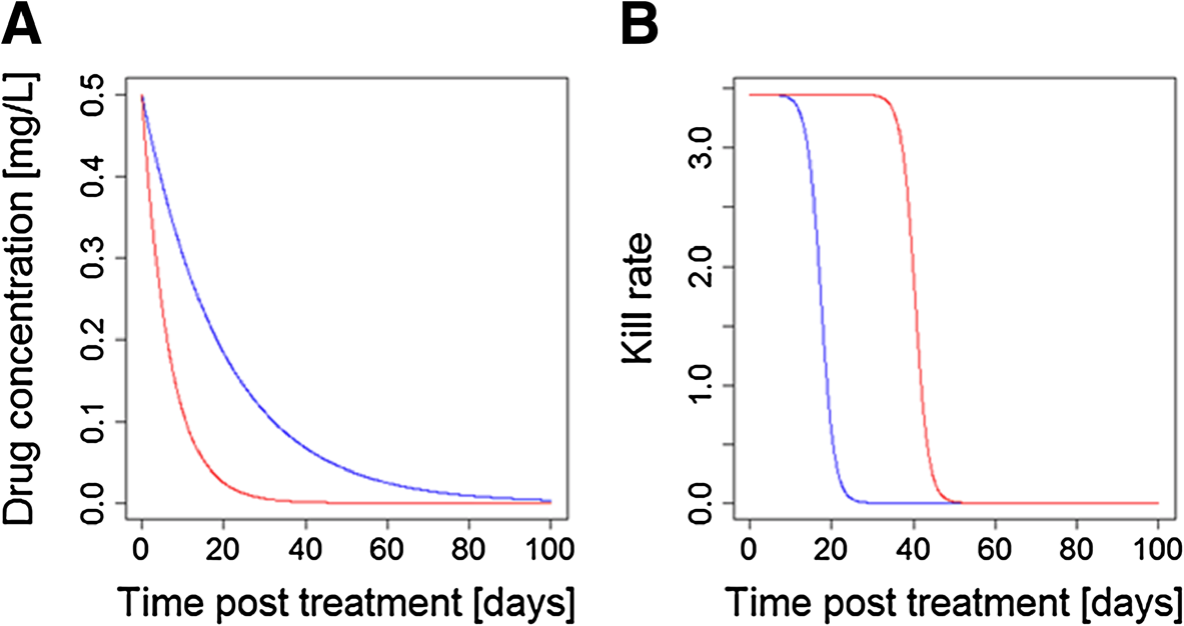
**How natural variation in PK/PD may undermine matched post-treatment drug activity profiles.** The two drugs have, on *average*, the same PK/PD parameters so are perfectly matched on *average*, c.f. Figure 3C. In these examples, natural variation around these mean PK/PD values results in one drug in the CT being exposed as a monotherapy for a significant period post-treatment. These illustrative differences reflect variation in single parameters: mismatches may become much larger once simultaneously variation in all PK/PD parameters is included. **(A)** An example of the impacts of differences in human PK, elimination rate; the red drug is eliminated by this patient 50% faster than the average while the blue drug is eliminated 50% slower than the average. **(B)** How variation in parasites PD parameters affect these profiles: the patient has the same PK for each drug (so concentration profiles post-treatment for both drugs are identical) but the parasites inoculated into the patient differ in their sensitivity to the drugs: their 10-fold higher resistance (IC50) to the blue drug means the red one is effectively a monotherapy for a significant period of time post-treatment. [Figure 4 was constructed using simple PK/PD models and their corresponding equations [22,25]. Parameter values for the two drugs are as follows: Dose is 75 mg/kg; volume of distribution is 150 L/kg; elimination rate per day is 0.05 for ‘blue’ and 0.15 for ‘red’ (equivalent to half-lives of 13.8 and 4.6 days, respectively); maximal drug-killing rate per day (Vmax) is 3.45; IC50 is 0.088 mg/L for ‘blue’ and 0.0088 mg/L for ‘red’; slope of dose-response curve (n) is 6].

One key operational question in CT design is the extent to which parasites vary in their PD parameters such as IC50. A first step in investigating the likely effectiveness of CT design is to obtain a value for mean IC50 plus the nature and extent of variation around this mean [28]. The variation in IC50 values is critical as it determines the extent to which natural variation in PD will undermine an idealized matched profile. Balancing half-lives, dosages and drug sensitivity can only be plausibly done on the basis of their average values (as used in Figures 2 and 3); in practice, the strategy will be hindered by the huge natural variation that occurs in both PK and PD parameters. Even if it were possible to match drug profiles in an ‘ideal’ human, it would appear inevitable that natural variation in PK/PD parameters would cause a mismatch in individual patients’ post-treatment concentration and activity profiles and leave one drug persisting as a vulnerable monotherapy. The variation typically noted in PK and PD parameters is huge. The between-subject variability in human PK parameters is typically 30–50% [36] while variation in parasite isolates IC50 values typically vary 100 to 10,000 fold (see, for example, Figure 3 of [37]). Figure 4 illustrates how this variation may undermine matching. The natural variation in PK will be augmented by human ‘behavioural’ variation e.g. food intake, age, nutritional status and factors such as pregnancy (reviewed in [38]). If these factors differentially affect the individual drugs in the CT then mismatches may be widened. In order to avoid amplification of the already large between-subject variation in PK parameters clear recommendations must be given on covariates that affect PK such as co-administration of food intake and dose adjustments in children under the age of five or pregnant women. Implementing these recommendations is not straightforward because providing such advice must be balanced against the need for clear instructions on drug use in resource-poor regions where patients are often treated in the informal sector and may have only casual access to anti-malarials.

It is, therefore, inevitable that mismatch will arise in individual treatment and that mutual protection will be much less than anticipated. The extent to which this will undermine the advantages of CT is uncertain although recent PK/PD modelling suggests the selection pressures associated with this mismatch (the ‘windows of selection’) is likely to be much lower that currently thought [39].

Most arguments for matching half-lives are over-optimistic and simply show a variant of Figure 1B without considering how factors such as natural variation in PK/PD can affect this matching. In conclusion, it is only plausible to make approximate matches on the basis of half-live. Most drugs outside the artemisinin class have relatively long half-lives so could be approximately matched with the caveats listed above.

#### 3. Dosages and toxicity

There are two main types of toxicity associated with malaria drugs. Those which are dose- or concentration dependent (also referred to as ‘type A’) adverse drug reactions (ADRs), which are mostly predictable and consistent between patients because they are explained by the drug’s known pharmacological action. The second is ‘type B’ ADRs that are generally, or at least partially, dose- or concentration independent and which are largely unpredictable and dependent on an individual patient’s metabolism, immune system or genetics. One common example for anti-malarial drugs is glucose-6-phosphate dehydrogenase (G6PD) deficiency which mediates toxicity in drugs from at least two different classes, e.g. primaquine and dapsone (Table 1). It is relatively easy to cure malaria, but curing malaria without poisoning the patient is much more problematic. Guinea-Bissau (G-B) overcame its problem of chloroquine (CQ) resistance by simply doubling the dosage of CQ given to patients. This was an effective strategy, but raises safety concerns over toxicity (although adverse events were not observed in practice [40]) and to date no other country has followed this approach. One feature of current anti-malarial drugs is that their therapeutic index (TI; the ratio between a drug’s upper ‘toxic’ and lower ‘curative’ thresholds) is very narrow (between 1.5 and 3 [41]) and only artesunate (AS) and dihydroartemisinin (DHA) show a TI ≈ 5. This feature hinders attempts to balance doses to obtain a matched elimination profiles (see Figure 3C) because dosing regimens with doses exceeding the upper threshold of the target dose range are likely to increase the number of patients experiencing type A reactions.

Combining two drugs always increases the risk of toxicity, but it is important to quantify this increased risk and to describe how it can be mitigated. The obvious assumption is that risk of toxicity to the CT is additive (i.e. the product of the risks of each drug given individually) but, in principle, toxicity could be synergistic (i.e. the risk is greater than twice the product) or may be antagonistic if the presence of a drug decreases the risk of toxicity caused by the other drug. Predicting synergy or otherwise in drug toxicity is problematic. Laboratory studies are vital to estimate this and, for example, animal studies showed that the teratogenic potential of the combination sulphadoxine-pyrimethamine (SP) was two-fold higher than expected from the drugs individually (for review see e.g. [45]). Synergy may plausibly occur if drugs share a common toxicity mechanism e.g. in G6PD-deficient patient, so such drugs should probably not be combined. Synergy could also arise if drugs share the same metabolic pathways for elimination because competition for metabolizing enzymes could extend the elimination half-life and hence the areas under the drug concentration curve. Similarly, competition for plasma protein binding sites could result in higher plasma drug levels for both drugs. Note that both increased half-lives and increased concentrations would actually increase the effectiveness of the drugs so drug effectiveness and toxicity may well be positively correlated.

**Figure 5:**
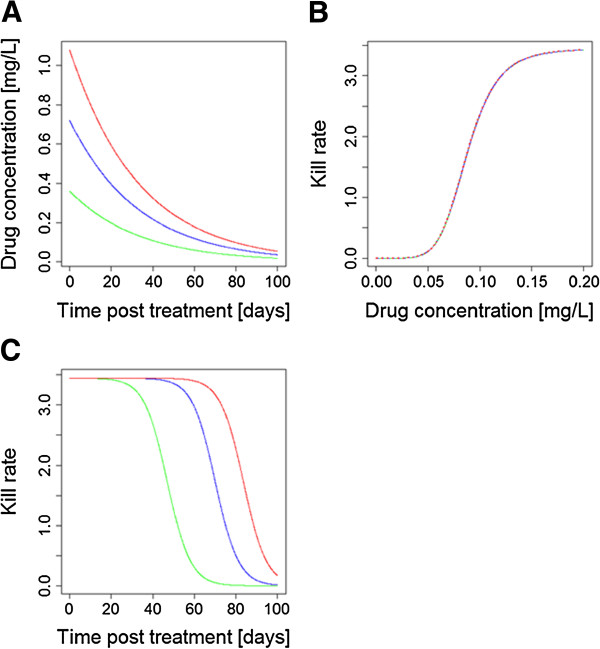
**The Law of Diminishing Returns when increasing drug dosages.** This example is based on piperaquine using PK/PD parameters from Table 1 of Winter & Hastings [25]. **(A)** The drug concentrations post-treatment: the green line is the standard dose of three daily doses of 18 mg/kg given as a single dose of 54 mg/kg (for illustrative purposes), the blue line is a double dose (108 mg/kg), and the red line is a triple dose (162 mg/kg). **(B)** The Michaelis-Menton relationship between drug concentration and anti-malarial activity. **(C)** The activity profiles post-treatment of the three different doses, obtained by multiplying the drug concentrations by their killing rate. Doubling the dose gave only an extra 49% area under the drug killing curve while tripling the dose gave only an increase of 19% compared to the double dose. [Figure 5 was constructed using simple PK/PD models and their corresponding equations [26,29]. Parameter values for the three drugs are as follows: Dose is 54 mg/kg for ‘green’, 108 mg/kg for ‘blue’ and 162 mg/kg for ‘red’; volume of distribution is150 L/kg; elimination rate per day is 0.03 (equivalent to half-life of 23.1 days); maximal drug-killing rate per day (Vmax) is 3.45; IC50 is 0.088 mg/L; slope of dose-response curve (n) is 6].

One operational question in CT design is the extent to which doses of the constituent drugs can be reduced to lower the risk of ADRs; the reduced effectiveness of each drug (caused by its lower dose) would be offset by the presence of its partner drugs in the CT. For example, in both animal and human *Plasmodium* infections, pyrimethamine and sulphadoxine administered together are curative at one-eighth the dose of either used alone [45]. An important factor in considering this strategy is that increasing individual drug dosages display a law of diminishing returns in terms of anti-malarial activity (Figure 5) while increasing doses often increase the risk of toxicity in an additive manner. Development of the promising anti-malarial drug combination chlorproguanil-dapsone (‘Lapdap’) plus AS was discontinued after the drug showed G6PD-associated toxicity in Phase 3 clinical trials [46]. It is believed that dapsone was responsible for the toxicity and it remains a possibility that the CT could be re-evaluated by reducing dapsone dosages to reduce toxicity and relying on artesunate to offset the reduced therapeutic effects of the lower dapsone dose. Such a proposal would probably have to initially rest on accurate PK/PD modelling of its efficacy, which could simultaneously investigate how the reduced-dose CT would by threatened by, and possibly drive, the spread of resistance [28].

#### 4. Do the drugs have independent pharmacodynamics?

Drugs within a CT may act additively, synergistically or antagonistically in their ability to kill malaria parasites. Chou [47] noted that the definition of synergy is fraught with difficulty and misunderstanding and readers should view this paper for a full discussion. A simple intuitive approach can be used in this context where synergy is an action greater than the sum of the two drugs used separately and antagonism occurs where the two drugs have less anti-malarial activity than would be expected from their individual activities. Synergy/antagonism in PD effects is often detected *in vitro* through construction of isobolograms, which quantify how parasite killing or growth inhibition depends on the concentrations of both drugs in a combination [48-51].

It would be expected intuitively that drugs with the same mode of action would be largely antagonistic. For example, if a drug completely blocks the haem polymerization pathway then there would appear little point in combining it with another haem-inhibiting drugs and blocking it twice. Similarly artemisinins are converted to their active metabolite DHA in vivo, and presumably both forms have the same target; it would be probably wrong to regard both forms as having independent PD (see discussion in [28]).

An alternative ‘intuitive’ expectation is that using two drugs with the same mode of action would be additive and, crudely, would have double the effect of either one alone; this is consistent with the fact that all anti-malarial drugs so far deployed have had their dosages increased to improve effectiveness. In fact, the reverse is probably true: PK/PD arguments suggest that increasing dosages will suffer from a law of diminishing returns if drugs share the same mode of action; see Figure 5. In essence, increasing the dose of the same drug extends the duration of effect, rather than it magnitude (Figure 5) if two drugs in a CT share the same PD it is likely that the drug with the longest post-treatment activity (see Figure 3) will be the main determinant of therapeutic outcome and its partner’s contribution may be small to negligible. Diminished returns may still be operationally useful, for example the additional 49% parasite killing associated with the example on Figure 5 may still be enough to restore drug efficacy, but it does not necessarily represent best use of drugs within a CT, hence the usual advice to avoid combining drugs with the same mode of actions (e.g. [32,52]). The law of diminishing returns may lead to a practical problem of a potential single dose cure [17,32]. While a single dose regimen might be a real game changer from a patient adherence perspective, the single dose needed to achieve the same extent of killing might, for example, lead to an inacceptable high Cmax, or the physical tablet size might make it impossible for patients to swallow it.

Combining drugs with additive or synergistic action should also increase parasite clearance post-treatment and hence may speed the resolution of symptoms. This is obviously desirable but much less important, certainly from a resistance standpoint, than whether a patient is actually cured. Clearance post-treatment is also complicated in anti-malarials because it is mainly determined by the fastest acting drug, invariable an artemisinin, in ACT. Artemisinins have a short half-live and show stage specific killing so clearance rate is also affected by the malaria parasite stages that predominate at the time of treatment. Clearance is also complicated by patient immune status [53,54], with immune patients clearing parasites more rapidly.

One potential drawback of synergy is that the inter-dependency between the drugs means that if resistance evolves to one individual component then the CT may start to fail. The best-known example is SP where early stages of resistance arise through mutations in the *P. falciparum* dihydrofolate reductase gene (*pfdhfr*) that encodes resistance to pyrimethamine. Sulphadoxine is unable to clear infections unaided by pyrimethamine and the CT as a whole started to fail once resistance to pyrimethamine started to evolve [55,56]. The theoretical basis for CT rests on the assumption that mutations in two or more genes are required to encode resistance to the CT. SP fails this design principle and hence the malaria community does not generally regard SP as a ‘true’ CT because, operationally, it behaves as monotherapy with mutations in a single gene capable of encoding resistance to treatment. The SP example illustrates a very important and often overlooked design principle: synergy between drugs in a CT is obviously beneficial but should not be used a reason to reduce individual drug dosages in the CT, except as a strategy to reduce concerns over toxicity (see above). Ideally each drug should be deployed at dosages that would be required for it to be effective as monotherapy so that the CT remains effective even when resistance is present to one of the components.

#### 5. Do the drugs have independent pharmacokinetics?

Pharmacokinetic processes are often non-independent and/or saturable so one consideration of CT design is the extent to which individual drug PK are affected by co-administration with their partner drugs. For example, lumefantrine (LF) absorption appears to saturate, so lower doses given more often is more effective [57]; drugs sharing the same absorption route as LF could compete for absorption and hence be antagonistic which would reduce their efficacy within a CT. If drugs share the same conversion or elimination pathways post-absorption then their actions could become non-additive but in unpredictable ways. For example, if conversion to an active form is impaired by the presence of a partner drug, and the unconverted form is eliminated while awaiting conversion, then drug PK may be antagonistic. Conversely, if the same elimination pathway saturates for both drugs, and both parent forms are active, then drug half-lives may be extended and anti-malarial synergy may arise.

There appears to be little literature on the interactions between PK of anti-malarial drugs. However, the presence and importance of PK drug interactions is demonstrated by the much better characterized examples of interactions between drugs co-administered to treat different diseases. A recent review by Sousa *et al.*[58] stated that “Rifampicin, a standard component of combination regimens for treating TB, has a great influence on the bioavailability and the efficacy of several anti-malarial drugs, not only because of the inhibition of Phase I and II enzymes of hepatic metabolism, but also because of its effect on drug absorption and distribution. It induces almost all cytochrome P450 (CYP) enzymes, it inhibits *N*-acetyltransferases and it alters the expression of membrane transporters.” Malaria and HIV are co-endemic and there are detailed examples of how co-administration of anti-malarials and anti-retrovirals affects each other’s PK, many of which can be explained by the metabolic properties (i.e. induction or inhibition) of the co-administered drugs (recently reviewed by [59]). One class of anti-HIV drug are the protease inhibitors (PIs) which tend to increase exposure (defined as area under the plasma concentration–time curve and/or maximum concentration; for details see Tables 1 and 2 in [59]) of LF and decrease the exposures of artemether (AM) and DHA. Another class, the non-nucleoside reverse transcriptase inhibitors (NNRTIs), tend to decrease the exposures of AM, DHA and LF, when co-administered with AM-LF [59]. Fewer studies characterized the effects of PIs or NNRTIs on AS combinations, where nevirapine (a NNRTI) increased AS exposure and ritonavir (a PI) decreased DHA exposure. These interactions may be mutual: AM-LF or AS combinations had little effect on the PK of HIV-anti-retrovirals, although AM-LF resulted in decreased nevirapine exposure and pyronaridine-AS increased ritonavir exposure [59]. It is therefore certainly plausible that drugs in an anti-malarial CT can induce or inhibit each other’s metabolism. Artemisinins drugs are potential inducers of CYP enzymes, and the most inducible are CYP2B6 and CYP3A4, which are believed to be the main enzymes involved in the auto-induction of artemisinin drugs [60]. In a pooled PK analysis the onset of auto-induction for artemisinin was found to be very rapid, e.g. 8 h after the first dose [61]. This implies that a single dose of artemisinin is capable of enzyme induction. The analysis suggested auto-induction has minor effects on the systemic clearance of artemisinin but results in a 13-fold decrease in its bioavailability. The metabolism of drugs is also affected by regional differences in the prevalence of anti-malarial drug-metabolizing enzyme polymorphisms (for a detailed discussion see [62]) which further complicate the link between drug dosage and subsequent anti-malarial drug concentration and treatment outcome.

 In essence, the consequences of PK interactions are difficult to predict and depend on the exact metabolic pathways and whether or not active metabolites contribute to parasite killing. One big advantage over the other factors enumerated here is that PK interactions can be measured in preclinical studies and clinical (Phase I) studies of CT development in healthy human volunteers. Pharmacokinetic parameters of anti-malarial drugs are known to be affected by malaria infections (e.g. quinine clearance is reduced in acute malaria, primarily as a result of disease-induced dysfunction in hepatic mixed-function oxidase activity [63]) so the Phase I study results are not definitive, but have the huge advantage of not requiring infected patients which would raise a whole series of ethical issues based around the rights of patients to receive current local best standard of care.

#### 6. Do the drugs share common mechanisms of cross resistance?

The benefits of using a CT rest on the parasite population having to evolve resistance to both drugs to survive treatment with the CT (see above). Intuitively, cross-resistance between the constituent drugs in a CT will undermine this effect and modelling shows that even small amounts of cross resistance may significantly reduce the expected therapeutic lifespan of both drugs [64]. The clearest example of cross-resistance in anti-malarials occurs in the antifolates class. The two best-known antifolate combinations are SP and chlorproguanil-dapsone. Sulphadoxine and dapsone both inhibit *P. falciparum* dihydropteroate synthase (PfDHPS) while pyrimethamine and chlorcycloguanil, the active metabolite of chlorproguanil, both inhibit *P. falciparum* dihydrofolate reductase (PfDHFR). Parasites carrying single-point mutations in the *pfdhfr* gene showed decreased sensitivity towards pyrimethamine, rising from 10-fold associated with a single mutations to 1,000-fold associated with a quadruple-mutant allele [65]. Similarly, parasites that have accumulated several mutations in the *pfdhps* gene display resistance towards sulphadoxine [66]. While the so-called ‘triple mutant’ with point mutations at codons 108, 51 and 59 of the *pfdhfr* gene is resistant against pyrimethamine and sensitive to chlorcycloguanil, the additional PfDHFR mutation at codon 164 renders chlorproguanil-dapsone ineffective [67]. The obvious question is the extent to which this paradigm of cross-resistance in antifolates is likely to extend to the other classes and it is useful, in this context to examine the lessons learnt from the evolution of insecticide resistance. Researchers in this field have noted a dichotomy in resistance mechanisms (e.g. [68]). ‘Target site’ resistance arises when mutations in the insecticide target site disrupt binding of the insecticide to its target, usually an enzyme. The second mechanism is ‘metabolic resistance’ where mutations disrupt the ability of an insecticide to reach, or accumulate in, its target site; typical mechanisms are metabolic detoxification of the insecticide or cell pumping mechanism that prevent insecticide accumulating at its target and these mechanism are usually associated with changes in expression levels of genes in the adenosine triphosphate-binding cassette (ABC) transporters and CYP gene families [69]. The main mechanism of resistance to anti-malarial antifolates appears to be target site resistance in the *pfdhfr* and *pfdhps* genes but known mechanisms of resistance to CQ, mefloquine (MQ) and LF are metabolic and involve mutations and/or copy number variation in the *P. falciparum* chloroquine resistance transporter (*pfcrt*) and multiple drug resistance protein-1 (*pfmdr1*) genes both of which encode cell membrane transporters. Cross resistance in this case is more likely to depend on the chemical structure and ionic charge of the drug than on its eventual target site [70]. This may explain why, for example, mutations in the *pfcrt* gene decrease resistance to LF while increasing resistance to CQ. Both drugs are in the same class (Table 1) but their structure means that they are recognized differentially by the cell transporter mechanism. Similarly Basco & Ringwald [71] demonstrated that piperaquine (PPQ) remains active against CQ-resistant parasites. These drugs are both 4-aminoquinolines (Table 1) and their close structural similarity suggests they would have the same mode of action, but these observations suggest there are different mechanisms of resistance, presumably through ‘metabolic resistance’, to the different drugs. In summary, many non-antifolate anti-malarial drugs disrupt the process of haem crystallization so target a physio-chemical process rather than having a specific enzyme target site. The absence of a definite, parasite-encoded target molecule for such drugs makes it highly likely that metabolic, rather than target-site, resistance is the main mechanisms of resistance which means the chances of cross-resistance, even within drugs in the same class, are greatly diminished.

Cross resistance can be quantified as a correlation between IC50 observed in field and/or laboratory isolates. As a recent example, Mu and colleagues [37] measured the *in vitro* drug sensitivity (i.e. IC50s) of 185 field isolates to seven drugs from four different classes. They reported the correlation between IC50s to different drugs (their Figure 3). The results were interesting: correlations were generally weak with correlation coefficients typically around -0.2 to 0.4, but were not closely dependent on the class of origin of the drugs. In fact, the two drugs with the strongest correlation in IC50 came from two separate classes, the artemisinin derivative DHA, and the arylamino alcohol MQ (Table 1). In addition, there may be far less diversity in resistance mechanisms than might be anticipated: Yuan *et al.*[72] screened a library of 2,811 chemical compounds for anti-malarial activity, identified 32 highly active compounds and then tested them in 61 parasite isolates. They found that three resistance loci, *pfcrt*, *pfdhfr*, *pfmdr1* were involved in 96% of cases where there was significant variation in isolate drug sensitivity.

One caveat associated with such data obtained from field isolates is that the IC50s may reflect the effects of standing genetic variation at many loci as a kind of ‘baseline’ drug sensitivity before high-level resistance arises through individual mutations with large effects. There is no guarantee that the same correlations in IC50s noted from field isolates will be associated with individual mutations that have major effects of drug sensitivity. Data are limited on this and, to date, the only clear example is that of the *pfcrt *K76T mutation which increases resistance to CQ but appears to only slightly increase sensitivity to LF [43]. See Ecker and colleagues [73] for a more detailed discussion of how single mutations affect drug sensitivity in general and in the best known case of *pfcrt* and CQ resistance [74].

It is often recommended that combining drugs from same class into a CT should be avoided (e.g. [32,41]) and the usual reasoning is because a mutation may occur that encode cross-resistance to members of the class (and thence the two drugs in the CT). This would be undesirable but, with the exception of antifolates, such mutations have not been observed. Far more serious is the fact that drugs in the same class probably share the same mechanism of parasites killing and, possibly, mechanisms of human toxicity; these latter two factors are likely to be of more immediate concern in CT design than the longer term threat of resistance.

**Table 1:** Currently available classes of anti-malarial drugs

**Drug class**	**Example drugs**	**Comments**
Artemisinins (or artemisinin derivatives)	Artesunate, artemether and dihydroartemisinin	The most widely used of the anti-malarial drugs with very short half-lives. These are sub-curative in standard 3 day regimens if used as monotherapies
Antifolates	Pyrimethamine, chlorproguanil, proguanil, sulphadoxine and dapsone	The combination sulphadoxine-pyrimethamine (SP; also known by its trade name ‘Fansidar’) is widely used for therapy. Both constituents have long half-lives so it was given as a single-dose regimen but resistance quickly evolved. Its use is now primarily restricted to treatment/prophylaxis in intermittent treatment programmes
4-aminoquinolines	Chloroquine, amodiaquine, piperaquine, pyronaridine and naphthoquine	Chloroquine was used in huge quantities as a monotherapy for over 30 years. Resistance occurred only infrequently and Africa never developed its own resistance instead it was aquired by immigrations from South-East Asia [42].
Arylamino alcohols	Quinine, mefloquine, lumefantrine and halofantrine	Quinine was the first anti-malarial to be identified. A long treatment duration and its safety profile means it is now mainly used in early pregnancy or as a (parenteral) second-line treatment either alone or in combination in uncomplicated or severe malaria. Lumefantrine with artemether is currently the most widely used anti-malarial combination therapy; it has low-level antagonistic resistance with chloroquine [43].
Naphthalenes	Atovaquone	Atovaquone is active against hepatic and asexual stages but resistance arises spontaneously at very high rates. Has synergistic pharmacodynamics when combined with proguanil, resistance no longer occurs at high rates and the combination therapy widely used as prophylaxis under the trade-name ‘Malarone’. Can also be used curatively but high cost restricts its deployment in resource-poor health services.
8-aminoquinolines	Primaquine and tafenoquine	These drugs affect hepatic and transmission stages but do not affect the pathogenic asexual stages of the plasmodium cycle so are not routinely uses to cure acute infections. Both are toxic in glucose-6-phosphate dehydrogenase deficient patients [44]. Primaquine has a short half-life which reduces its therapeutic effectiveness but means concentration can be allowed to drop very rapidly in patients identified with adverse reactions.
Antibiotics	Tetracycline	These drugs do have activity against the asexual stages but their slow speed of action precludes their use as therapeutics

**Table 2:** An ideal Target Product Profile (TPP) for an anti-malarial combination therapy

**Property**		**Attribute**	**Reference**
Formulation & dose	Single-dose treatment regimen	Desirable	[17,32]
	Stable	Critical	[32]
	Fixed-dose in a single formulation	Desirable	[32]
	Orally, rectally and parentally applicable	Desirable	[32]
	Dose of each drug high enough so that it will remain effective even if resistance is present to the other drug	Critical	This manuscript KPC#4
Mode of action	Effective against all stages of parasite development in the human host	Desirable	[32]
	Active against hypnozoites and able to prevent relapse	Desirable	[17]
	Transmission-blocking activity	Desirable	[17]
	Robust to the evolution of resistance	Critical	[32]
	Independent, or preferably synergistic, mode of action of drugs	Desirable	[32]; this manuscript KPC#4
	Different metabolic target(s) of drug action	Desirable/Critical	This manuscript KPC#4
	Negative patterns of cross resistance	Desirable	This manuscript KPC#6
Pharmacokinetics & pharmacodynamics (PK/PD)	Elimination half-lives of drugs should be approximately matched	Desirable	[32,33]
	The post-treatment drug activity profiles (based on elimination half-lives, dosages and drug sensitivity) should be matched	Critical	This manuscript KPC#1 (Figures 2 & 3)
	Low levels of inter-individual PK/PD variation to minimise drug activity profile mismatch in individual infections	Desirable	This manuscript KPC#2 (Figure 4)
	Extended period of chemoprophylaxis post-treatment	Desirable	[15,17]
	Predictable metabolism via non polymorphic enzymes	Desirable	This manuscript KPC#5
	No pharmacokinetic drug-drug interaction	Desirable	This manuscript KPC#5
Efficacy & safety	Large therapeutic index	Desirable	This manuscript KPC#3
	Toxicity of drugs should be additive or antagonistic	Desirable	This manuscript KPC#3
	Drug conversion and elimination should not share same metabolic pathway	Desirable	This manuscript KPC#3
	Dissimilar type B adverse drug reaction profiles	Desirable	This manuscript KPC#3
	Safe and well-tolerated	Critical	[32]
	Efficacious and effective	Critical	[32]
Cost	Affordable/cheap	Critical	[17,32]

KPC: key pharmacological consideration.

### Target product profiles for combination therapies

The development of new products may be informed by clearly identifying the desired properties of the final product, the Target Product Profile (TPP). One problem with TPPs is that they often constitute an idealized, but often unattainable, ‘wish list’ of what is required of a product; with this caveat, such a TPP is presented in Table 2 which builds on previous work presented by Kremsner and Krishna [32] and Burrows *et al.*[17] by incorporating the pharmacological considerations discussed above. There is unlikely to be a CT that fulfils all these desirable properties so the main operational problem is to trade-off the different characteristics offered by different formulations of CTs. This review has attempted to identify and quantify several key design principles in order to facilitate choice of CT design in a real world where no perfect CT is likely to exist in the medium to long term. The PK/PD arguments discussed above suggest the TPP for an ideal CT should include the following properties: the two drugs should act synergistically, have independent PK, have independent actions on toxicity, have negative patterns of cross resistance and have post-treatment drug activity profiles that can be matched. Importantly all these criteria are relatively easy to measure in culture (drug PD synergy, basal levels of cross resistance) or in preclinical work in animals (toxicity) and early clinical work in humans (PK, toxicity).

The problem is that there are only a limited number of existing forms of anti-malarials and design of new CTs may not be able to meet this TPP in the near future. A CT may not be ideal, but may still be useful because, importantly, even a CT that falls well outside that TPP can still be highly beneficial as the following success story illustrates; following Chou [47], the codes A and B are used for the two drugs. Drug A was failing badly, so was combined with Drug B. The match to the TPP was weak: high levels of cross resistance occurred, toxicity was additive, and drug activity follows a law of diminishing returns although and, more encouragingly, half-lives were matched. Drug A was CQ and Drug B was also CQ: as described above, Guinea-Bissau circumvented its problem of CQ resistance by the simple (although potentially toxic) expedient of doubling the dose of CQ given to patients to get a highly effective ‘CT’ [75]. This perfectly illustrates that it is possible to get a highly effective ‘CT’, capable of overcoming even high levels of resistance, by combining drugs within the same class even with 100% levels of cross resistance. The G-B story thereby illustrates an important design principle: that desirable and undesirable factors in a TPP can be listed but the ultimate test must be how a CT performs in the clinic. The CQ + CQ ‘CT’ violates all of the principles discussed above but its increased efficacy appears sufficient to eliminate parasites which are resistant to standard doses of CQ. The reason the G-B policy has not been widely copied is potential toxicity. The TIs of anti-malarials are relatively small (see above) and there is the additional operational requirement that drugs be deployed to dose according to weight/age/height bands within which heavy patients receive relatively low doses and light people receive higher, potentially toxic doses [30], and it is easy to see why policy makers are reluctant to follow the G-B strategy. The primary requirement is therefore that the two drugs are antagonistic, or at least not synergistic, in causing toxicity. The biggest operational decision in CT design is to decide whether to use the maximum dosages of each drug in a CT for the largest clinical effect and long-term robustness against resistance, or whether to reduce the dosages to reduce the risk of toxicity and hence maximize the short-term objective of ensuring safety and clinical approval.

One obvious question is whether triple- or even quadruple-combinations (as used to treat TB and HIV) could help meet the criteria of TPP. One obvious benefit for anti-malarials is that adding another longer half-life drug would partially remove the currently very large mismatch in periods of killing between the artemisinins and their typical partner drugs. In general, the more components, the more clinically effective the treatment is likely to be (for the reasons outlined above), but the obvious drawbacks are the increased cost, the possibility of more complicated regimes (which can affect patient adherence) and the increased risks of toxicity. The rationale design of such triple- or quadruple combinations can be guided by the design principles outlined above. One example of a currently-proposed triple combination anti-malarial is to add primaquine (PQ) to current ACT [76]. The important feature of these combinations is their mutually exclusive PD: ACT targets the asexual and early stage gametocytes while PQ targets mature gametocytes. This means that arguments based on matching PK/killing and independent/synergistic PD can be ignored and the combination evaluated on the basis of drug interaction in toxicity, interference between individual drugs’ PK and, possibly, mechanism of cross-resistance. These factors can then be weighed against the value of PQ in reducing malaria transmission.

## Conclusions

Maintaining a pipeline of effective anti-malarial drugs is a public health priority clearly recognized by the international community which supports the Medicines for Malaria Venture (MMV) to co-ordinate the research and development (R&D) pipeline of such drugs. The pipeline is relatively healthy [15] but the drug development process is slow and inherently unpredictable. MMV have a strategy for prioritizing development [17,77] but this unpredictability also places a responsibility on the R&D community for good stewardship and use of existing and newly-developed drugs. This is mainly achieved through their deployment as CTs to minimize the risk of resistance arising, and to maintain their long-term effectiveness. In most models of drug resistance, the ‘resistance’ trait is modelled as a purely parasite trait that determines whether or not the infection will be cleared by drug treatment. A PK/PD mechanism-based approach explicitly recognizes that drug ‘resistance’ (usually expressed as an increased IC50) is only one of a suite of pharmacological parameters (12 parameters for two-drug CTs) that determine a patient’s therapeutic outcome. In essence, it is necessary to understand how CTs successfully clear infections before starting to understand how, why and when individual patients fail treatment. Placing resistance in this context therefore reveals the pharmacological principles that determine what makes a ‘good’ CT and how the threat of resistance can be minimized in this context. It is argued herein that adopting a rational and objective method to simulate CT drug effectiveness using PK/PD principles can play a valuable role in this process.

## Abbreviations

ABC: Adenosine triphosphate-binding cassette; ACT: Artemisinin-based combination therapy; ADR: Adverse drug reaction; AM: Artemether; AS: Artesunate; AUC: Area under the curve; Cmax: Maximum concentration; CT: Combination therapy; CQ: Chloroquine; CYP: Cytochrome P450; DHA: Dihydroartemisinin; G6PD: Glucose-6-phosphate dehydrogenase; G-B: Guinea-Bissau; HIV: Human immunodeficiency virus; IC50: Drug concentration producing half the desired effect; LF: lumefantrine; MIC: Minimum inhibitory concentration; MMV: Medicines against Malaria Venture; MQ: Mefloquine; NNRTI: Non-nucleoside reverse transcriptase inhibitor; n: Slope of dose-response curve; P: *Plasmodium*; PD: Pharmacodynamics; pfcrt: *Plasmodium falciparum* chloroquine resistance transporter gene; PfDHFR: *Plasmodium falciparum* dihydrofolate reductase; pfdhfr: *Plasmodium falciparum* dihydrofolate reductase gene; PfDHPS: *Plasmodium falciparum* dihydropteroate synthase; pfdhps: *Plasmodium falciparum* dihydropteroate synthase gene; pfdmdr1: *Plasmodium falciparum* multi-drug resistant protein 1 gene; PI: Protease inhibitor; PK: Pharmacokinetics; PPQ: Piperaquine; PQ: Primaquine; R: Resistant; R&D: Research and development; S: Sensitive; SP: Sulphadoxine-pyrimethamine; TB: Tuberculosis; TI: Therapeutic index; TTP: Target product profile; Vmax: Maximal drug-killing rate per day.

## Competing interests

The authors have no competing interests.

## Authors’ contributions

IMH and EMH have been involved in drafting the manuscript and revising it critically for important intellectual content. Both have given final approval of the version to be published.
